# Idiopathic venous thromboembolic disease is associated with a poorer prognosis from subsequent malignancy

**DOI:** 10.1038/sj.bjc.6605210

**Published:** 2009-08-04

**Authors:** A Jones, D L Stockton, A J Simpson, J T Murchison

**Affiliations:** 1 Department of Respiratory Medicine, Royal Infirmary of Edinburgh, Little France, Edinburgh, EH16 4SA, UK; 2 Scottish Public Health Observatory, Information Services (ISD), NHS Scotland, 1 South Gyle Crescent, Edinburgh, EH12 9EB, UK; 3 Department of Radiology, Royal Infirmary of Edinburgh, Little France, Edinburgh, EH16 4SA, UK

**Keywords:** idiopathic venous thromboembolism, malignancy, prognosis

## Abstract

**Methods:**

We carried out a retrospective study of prognosis in Scottish patients diagnosed with cancer within 5 years after a venous thromboembolism (VTE).

**Results and conclusions:**

Prognosis was significantly poorer if a VTE occurred up to 2 years before cancer diagnosis, most notably if the cancer was diagnosed in the 6 months after a VTE.

Trousseau first described an association between malignancy and venous thromboembolism (VTE) more than a 100 years ago (Trousseau, 1872), since which time it has become clear that malignancy is a risk factor for VTE. More recently, several large retrospective studies have shown an increased risk of cancer after a diagnosis of idiopathic VTE, with standardised incidence ratios of 3–4.2 within the first 6 months (Nordstrom *et al*, 1994; Sorensen *et al*, 1998; Murchison *et al*, 2004). The length of time for which increased risk can be shown varies and we have shown a persistent, although declining, risk up to 2 years from VTE diagnosis (Murchison *et al*, 2004). Those with secondary VTE do not seem to be at a similar risk of future malignancy (Prandoni *et al*, 1992; Bastounis *et al*, 1996). In view of this increased risk, there has been an interest in screening patients with idiopathic VTE for occult malignancy. Initial retrospective studies in patients with idiopathic deep vein thrombosis (DVT) suggested that most occult malignancy could be detected by routine evaluation (Nordstrom *et al*, 1994; Cornuz *et al*, 1996). However, more recent prospective studies advocate a more intensive investigative approach (Monreal *et al*, 2004; Piccioli *et al*, 2004).

Relatively little is known about the impact that a preceding VTE has on the prognosis of subsequent malignancy, and this may well inform the screening debate. A Danish registry study reported that patients diagnosed with cancer during their admission for VTE had a 1-year survival rate of 12% compared with 36% in the control group of cancer patients without preceding VTE; 1-year survival rates were also poorer in those diagnosed with cancer in the year after a VTE (Sorensen *et al*, 2000).

Following on from previous work, we examined the prognosis of Scottish patients in whom malignancy was diagnosed at the time of, or in the 5 years after a VTE.

## Methods

The Information Services Division (ISD) of the National Health Service in Scotland contains linked, anonymised information (using probability matching) on all Scottish hospital in-patient discharges, death records and cancer registry records from 1981 to 2005. For this study, all records with a diagnosis of DVT or pulmonary embolism between 1982 and 2000 were selected from the linked, non-identifiable database, giving a population-based database covering 19 years.

To reduce the number of patients with secondary VTE, those who had undergone surgery in the preceding 6 weeks were excluded, as were pregnant women. Owing to difficulties in coding, we were unable to exclude those with hereditary thrombophilias. Records without a Scottish postcode were excluded.

Cancers were classified as occurring after a VTE if they represented a first diagnosis of malignancy (excluding non-melanomatous skin cancers) in the 5 years after a VTE.

Patients with a previous diagnosis of malignancy were excluded to avoid cancer itself being a confounding factor for subsequent malignancy. Cancer registrations derived solely from death certificates were excluded because of a lack of survival information. Follow-up for the occurrence of cancer was carried out from the initial diagnosis of VTE until the end of 2000, allowing a 5-year survival analysis of all cancer patients after diagnosis up to the end of 2005.

Kaplan–Meier survival analysis was used to obtain estimates of crude survival at 1 and 5 years after cancer diagnosis by cancer site and timing of VTE. Multivariate Cox's proportional hazard models (Collett, 1994) were used to assess the impact of VTE on survival for each cancer site separately. Adjustment was made for age (<50, 50–59, 60–69, 70–79, 80+ years), sex, period of diagnosis (1986–90, 1991–95, 1996–2000) and deprivation decile (Carstairs and Morris, 1991). Patients without a VTE in the 5 years before malignancy served as the reference group for multivariate analyses. The end point was death from any cause.

## Results

We identified 4322 patients diagnosed with VTE, who were then diagnosed with a first malignancy in the subsequent 5 years. The 299 714 patients diagnosed with malignancy in the same time period, but who had not been diagnosed with a VTE in the preceding 5 years, served as the reference group. The breakdown of these figures by cancer site and timing of cancer diagnosis is given in Table 1. Among those with VTE and malignancy, the most common cancer sites overall were the lung, colon, prostate and breast. Patients with a VTE up to 2 years before a diagnosis of malignancy had a poorer prognosis than those without a preceding VTE, as detailed in Table 2. Across all the cancer sites examined and in the group as a whole, prognosis was particularly poor if the VTE occurred at the time of, or in the 6 months before, diagnosis of malignancy, with a hazard ratio (HR) of 2.48 (*P*<0.001) for all cancers combined. Within this group, malignancies of the cervix, uterus, pancreas, rectum and breast had the poorest prognosis comparatively, with HRs >2.5. For all cancers combined, the HR remained elevated at 1.21 (*P*=0.002) and 1.26 (*P*<0.001), respectively, for those with a VTE 6–12 months and 1–2 years before cancer diagnosis.

## Discussion

This study examined the survival of over 4000 patients diagnosed with cancer at the same time as, or in the 5 years after, a diagnosis of VTE, and compared it with cancer patients who did not have VTE at presentation or in the preceding 5 years. Those diagnosed with cancer 0–6 months after a diagnosis of VTE have a significantly poorer prognosis, and this remains so for patients diagnosed with VTE up to 2 years before cancer diagnosis. The crude 1-year survival for all cancers combined was 19% for those diagnosed at the time of, or in the 6 months after, VTE. This compares with a 54% 1-year survival for all cancers in the reference group (data not shown). Our findings are in keeping with earlier work showing a mortality ratio of 2.46 at 1 year if VTE and cancer were diagnosed concurrently (Sorensen *et al*, 2000).

A limitation of our study is the lack of staging data, which ISD only began collecting for a limited number of cancers in 1997 and which were insufficient for analysis. Therefore, we cannot comment on the prevalence of metastatic disease at presentation, and the poor prognosis observed in those with VTE at this time or in the preceding 6 months may reflect a later stage at diagnosis. In a previous study, Sorensen *et al* did not match for stage, but did have staging data. They found a prevalence ratio of 1.26 for metastases in those diagnosed with VTE and malignancy concurrently, compared with controls (Sorensen *et al*, 2000). It is therefore likely that the extent of cancer at presentation, as measured by traditional staging methods, does not fully explain the poorer prognosis of these patients.

Morbidity and mortality directly caused by VTE itself are unlikely to be solely responsible, particularly as the prognosis remains poor for malignancies diagnosed up to 2 years after VTE. A complex interaction between coagulation and tumour cell biology exists and there is a growing understanding of the potential role of coagulation pathways in modulating tumour growth (Nash *et al*, 2001; Rickles *et al*, 2003; Prandoni *et al*, 2005). However, it is not clear whether hypercoagulability itself predisposes to more aggressive cancer, or whether it is simply an early manifestation of such a disease. The former possibility suggests a potential for modulating tumour growth using agents acting upon the coagulation cascade.

This is a retrospective study and as such relies on the accuracy of the databases. The accuracy of discharge coding in Scotland is estimated at ∼90% (ISD Scotland, 2007); the quality of cancer registry data is also high (Brewster *et al*, 2002) with mismatches in the linkage system occurring in <2% of cases (Kendrick and Clarke, 1993).

This study provides evidence that, for all cancers combined, a diagnosis of VTE in the 2 years before cancer diagnosis is associated with a poorer prognosis, particularly if VTE is diagnosed concurrently or in the 6 months before cancer diagnosis. In view of the poor prognosis experienced by these patients, extensive investigation for underlying malignancy may not yield a survival benefit, and additional studies are required to clarify this. Further work is required into the mechanisms behind this association and the possibility for treatment targeted at the coagulation process.

## Figures and Tables

**Table 1:** Number of patients available for analysis according to cancer site and time period from diagnosis of VTE to diagnosis of malignancy

	**Number of patients according to time period from diagnosis of VTE to diagnosis of malignancy**				
**Cancer site**	**0–6 months**	**>6–12 months**	**>1–2 years**	**>2–5 years**	**No VTE in 0–5 years**
Oesophagus	42	21	27	8	8,697
Stomach	143	20	38	20	13,247
Colon	307	43	81	29	25,801
Rectum	69	6	37	8	12,776
Pancreas	186	15	18	7	7,284
Lung	538	107	155	72	56,779
Breast	92	39	80	24	39,262
Cervix uteri	29	4	11	2	5,305
Corpus uteri	29	2	8	5	4,479
Ovary	177	10	19	11	7,115
Prostate	163	35	92	33	19,750
Kidney	72	6	21	8	5,453
Bladder	78	16	56	9	15,275
Non-Hodgkins lymphoma	121	12	21	12	9,117
Lymphoid leukaemia	35	10	13	4	3,532
All malignancies^a^	2,710	425	871	316	299,714

ICD=International Classification of Diseases; VTE=venous thromboembolism.

a All malignancies within range the ICD 10 C00-C96, excluding non-melanomatous skin cancer.

**Table 2:** Multivariate hazard ratios according to time period for each cancer site

	**Multivariate hazard ratio according to time period from diagnosis of VTE to diagnosis of malignancy**				
**Cancer site**	**Control group**	**0–6 months**	**>6–12 months**	**>1–2 years**	**>2–5 years**
Oesophagus	1.00	2.45 (1.78–3.38)^a^	2.50 (1.25–5.01)^b^	0.73 (0.44–1.21)	0.84 (0.54–1.31)
Stomach	1.00	2.35 (1.98–2.79)^a^	1.61 (1.02–2.52)^b^	1.61 (1.04–2.50)^b^	0.87 (0.61–1.23)
Colon	1.00	1.95 (1.73–2.21)^a^	1.23 (0.83–1.84)	1.41 (1.01–1.98)^b^	1.16 (0.91–1.47)
Rectum	1.00	2.72 (2.12–3.49)^a^	1.62 (0.73–3.62)	2.60 (1.17–5.80)^b^	1.08 (0.74–1.58)
Pancreas	1.00	3.23 (2.77–3.77)^a^	0.97 (0.46–2.04)	0.70 (0.41–1.19)	1.36 (0.85–2.20)
Lung	1.00	1.93 (1.77–2.12)^a^	1.05 (0.82–1.35)	1.08 (0.88–1.32)	0.88 (0.75–1.04)
Breast	1.00	2.61 (2.09–3.27)^a^	0.86 (0.50–1.49)	1.14 (0.78–1.68)	1.18 (0.90–1.55)
Cervix uteri	1.00	4.84 (3.22–7.29)^a^	4.03 (1.0–16.22)^b^	1.45 (0.54–3.89)	1.44 (0.75–2.77)
Corpus uteri	1.00	3.87 (2.62–5.72)^a^	1.38 (0.44–4.33)	0.43 (0.06–3.06)	2.51 (0.94–6.73)
Ovary	1.00	2.34 (1.99–2.75)^a^	1.30 (0.72–2.36)	1.22 (0.55–2.72)	1.17 (0.70–1.95)
Prostate	1.00	2.07 (1.75–2.45)^a^	1.14 (0.78–1.66)	0.71 (0.45–1.09)	1.46 (1.17–1.83)^a^
Kidney	1.00	2.00 (1.55–2.57)^a^	0.94 (0.47–1.89)	1.13 (0.42–3.03)	2.07 (1.32–3.26)^a^
Bladder	1.00	2.24 (1.74–2.88)^a^	1.40 (0.67–2.93)	1.39 (0.81–2.40)	1.12 (0.82–1.52)
Non-Hodgkins lymphoma	1.00	2.09 (1.72–2.54)^a^	1.36 (0.70–2.61)	1.24 (0.62–2.48)	1.07 (0.65–1.75)
Lymphoid leukaemia	1.00	1.61 (1.12–2.29)^b^	1.20 (0.45–3.20)	1.04 (0.54–2.01)	0.89 (0.49–1.62)
All malignancies^c^	**1.00**	2.48 (2.38–2.58) *P*<0.001	1.21 (1.07–1.37) *P*=0.002	1.26 (1.13–1.40) *P*<0.001	1.07 (0.99–1.15) *P*=0.079

ICD=International Classification of Diseases; VTE=venous thromboembolism.

Results quoted are hazard ratios with 95% lower and upper confidence intervals in brackets.

a Indicates a *P*-value of <0.001.

b Indicates a *P*-value of ⩽0.05.

c All malignancies within the range ICD 10 C00-C96, excluding non-melanomatous skin cancer.
